# Vaccination with attenuated *Salmonella enterica *Dublin expressing *E coli *O157:H7 outer membrane protein Intimin induces transient reduction of fecal shedding of *E coli *O157:H7 in cattle

**DOI:** 10.1186/1746-6148-6-35

**Published:** 2010-07-07

**Authors:** Sangeeta Khare, Walid Alali, Shuping Zhang, Doris Hunter, Roberta Pugh, Ferric C Fang, Stephen J Libby, L Garry Adams

**Affiliations:** 1 Department of Veterinary Pathobiology, College of Veterinary Medicine and Biomedical Sciences, Texas A&M University, College Station, TX 77843 USA; 2 Veterinary Integrative Biosciences, College of Veterinary Medicine and Biomedical Sciences, Texas A&M University, College Station, TX 77843 USA; 3 Department of Microbiology, North Carolina State University, Raleigh, NC 27695 USA; 4 University of Washington School of Medicine, Departments of Laboratory Medicine and Microbiology, Seattle, WA 98195 USA; 5 Division of Microbiology, National Center for Toxicological Research, US Food and Drug Administration, Jefferson, AR 72079 USA

## Abstract

**Background:**

*Escherichia coli *serogroup O157:H7 has emerged as an important zoonotic bacterial pathogen, causing a range of symptoms from self-limiting bloody diarrhea to severe hemorrhagic colitis and hemolytic-uremic syndrome in humans. Beef and dairy cattle are considered the most important animal reservoirs for this pathogen. One of the important virulence characteristics of *E. coli *O157:H7 is the *eaeA *gene encoding the 97 kDa surface protein intimin. Intimin is required for attachment and effacement during the interaction of enterohemorrhagic *E. coli *with human and bovine neonatal enterocytes. The present study was undertaken to test the hypothesis that an adaptive mucosal immune response directed against intimin will reduce or prevent enteric colonization and fecal shedding of *E. coli *O157:H7 in cattle.

**Results:**

Cattle were orally inoculated with either milk (control), milk with live attenuated *Salmonella enterica *serovar Dublin (vector), or milk with live attenuated recombinant *S*. Dublin expressing intimin (vaccinated) on days 0, 14 and 28. On day 98, all calves were challenged orally with *E. coli *O157:H7 to evaluate whether vaccination with the recombinant *S*. Dublin expressing intimin would reduce the level of *E. coli *O157:H7 fecal shedding.

During the first 28 days, vaccinated calves shed both the vector strain and the intimin-expressing *S*. Dublin strain at a similar level. The vector strain was shed for a significantly longer period as compared to the level of recombinant vaccine strain. Calves that received the intimin-expressed vaccine ceased shedding *S*. Dublin from day 28 to day 63. All calves were challenged with *E. coli *O157:H7 on day 98 to determine the effect on fecal shedding of *E. coli *O157:H7. The amount of *E. coli *O157:H7 in feces was measured for 30 days post-challenge. We observed a transient clearance of *E. coli *O157:H7 from the feces in the vaccinated calves. The magnitude of fecal *E. coli *O157:H7 shedding did not correlate with the presence of intimin-specific fecal IgA.

**Conclusion:**

Oral vaccination with live attenuated recombinant *S*. Dublin expressing intimin reduced enteric colonization and fecal shedding of *E. coli *O157:H7. However, the transient clearance of *E. coli *O157:H7 was not associated with an enhanced IgA-mediated mucosal immune response.

## Background

*Escherichia coli *serogroup O157:H7 (*E. coli *O157:H7) is a zoonotic bacterial pathogen that causes symptoms ranging from self-limiting bloody diarrhea to severe hemorrhagic colitis in humans [1,2]. *E. coli *O157:H7 infection can also cause extra-intestinal illness, most importantly hemolytic-uremic syndrome (HUS). The majority of *E. coli *O157:H7-associated fatalities results from renal failure, neurologic manifestations, or other complications of HUS [3-5]. *E. coli *O157:H7 is mainly a food borne pathogen. Beef and dairy cattle are considered to be the most important animal reservoirs of *E. coli *O157:H7 [6-12]. Transmission of *E. coli *O157:H7 by fecal contaminated water [13,14] is thought to be a major source of infection. Some person-to-person transmission has been also reported [15,16], but the main source of human infection with *E. coli *O157:H7 is contamination of food products.

The infective dose of *E. coli *O157:H7 is low for both calves and humans, in some cases approximately only 10^2 ^organisms are required to cause infection [17]. Neonatal calves are particularly susceptible to *E. coli *O157:H7, but adult cattle do not generally exhibit clinical signs following experimental or natural infection. Adult cattle typically continue to shed bacteria in their feces for weeks to months, or for the lifetime of the animal. Carcasses of non-colonized cattle have sometimes been found to contain *E. coli *O157:H7 in the abattoir, suggesting that cross-contamination during meat processing can be a major source of contamination of beef products and subsequent infection of humans [9].

One of the important virulence factors of *E. coli *O157:H7 is the *eaeA *gene that encodes the 97 kDa surface protein intimin. Intimin is required for *E. coli *O157:H7 colonization, the development of attaching and effacing epithelial lesions, and disease in neonatal calves, pigs, and mice [18]. Intimin-specific antiserum can block adherence of *E. coli *O157:H7 to HEp-2 cells in tissue culture [19]. Calves challenged with intimin-deficient mutant bacteria do not develop diarrhea or attaching/effacing lesions, nor are colonized to the same extent as animals infected with wild type or complemented mutant strains [20]. Earlier studies have proposed that mucosal IgA directed against intimin might serve an analogous function *in vivo *[21]. However, experimental challenge of cattle previously infected with *E. coli *O157:H7 has failed to demonstrate protective immune responses [22], perhaps because *E. coli *O157:H7 generate very low titers of specific mucosal IgA responses directed against intimin or other *E. coli *O157:H7 antigens [23]. *E. coli *O157:H7 colonization of mice can be reduced when the animals are fed recombinant tobacco expressing intimin [24]. It is suggested that intimin on the surface of EHEC would bind to nucleolin [25]. The present study was undertaken to test the hypothesis that a specific adaptive mucosal immune response directed against the surface antigen intimin might prevent or reduce the colonization of *E. coli *O157:H7 in cattle.

## Methods

### Cloning the *eaeA *gene into pRB3

The *eaeA *gene was amplified from pEB310 using primers SW20H3: 5'-CGCCCAAGCTTCGTTGTTAAGTCAATGG-3' and EaeA 3': 5'-CGCGGATCCAGTAGTAGATTTGATTATAAGAGG-3' by PCR and cloned into the *Hind*III/*Sma*I site of pRB3. Plasmid DNA was introduced into *S*. Dublin *aroA*::*tet *by electroporation. His-tagged EaeA was produced by cloning the coding region of *eaeA *into pET16b (Novagen, Gibbstown, NJ). Expression and purification of His-tagged EaeA on NTA-Nickel resin (Qiagen, Valencia, CA) was performed according to the manufacturer's instructions. His-tagged EaeA was concentrated and stored in 50 mM Tris-HCL 250 mM NaCl, 0.1 mM EDTA and 1 mM DTT.

### Identification of Salmonella- and *E. coli *O157:H7-free calves

Clinically healthy male Holstein/Friesian calves, aged 1 to 2 weeks, were obtained from a local supplier. The weight of the calves ranged between 40 and 45 kg. Animals were cared for according to the Association for Assessment and Accreditation of Laboratory Animal Care guidelines under the oversight of the Texas A&M University Institutional Animal Care and Use Committee AUP 2000-252. Calves were fed 2 liters of antibiotic-free whey-based milk replacer twice daily and given water *ad libitum*. Before being used for experiments, calves were clinically evaluated for fever and infection with *Salmonella *and *E. coli *0157:H7. The presence of *Salmonella *was evaluated by incubation of fecal samples in tetrathionate broth (Difco), followed by enrichment in Rappaport-Vassiliadis R10 broth (Difco), then by plating onto XLT-4 plates (BBL). All calves were free of *Salmonella *and *E. coli *O157:H7.

Calves were divided into 3 treatment groups. The control group consisted of 3 calves that were fed only milk replacer (950 ml) and 50 ml of inoculum buffer (a suspension of 5% magnesium trisilicate, 5% sodium bicarbonate, and 5% magnesium carbonate) on the inoculation days. The second group consisted of the vector group (4 animals). The calves in this group were inoculated with 10^10 ^colony forming units (CFU) of the *S*. Dublin *aroA *strain with the empty pRB3 vector suspended in 50 ml of inoculum buffer in milk replacer. The third group (hereafter called the vaccinated group) consisted of 5 calves inoculated with 10^9^-10^10 ^CFU *S*. Dublin *aroA *pRB3::*eaeA *suspended in 50 ml of inoculum buffer in milk replacer.

### Vaccination of calves with *S*. Dublin strains

Overnight cultures were grown in LB broth, and the optical density at 600 nm was determined. A volume containing the desired quantity of bacteria was added to 50 ml inoculum buffer. The inoculum was added to 950 ml of milk replacer and used to orally inoculate calves on days 0, 14 and 28. For all experiments, the bacterial titer of the inoculum was determined by plating serial dilutions onto LB agar plates, incubating plates overnight at 37°C, and enumerating the colonies.

### Challenge of calves with virulent *E. coli *O157:H7 strain 86-24

All calves were challenged orally with 10^10 ^CFU *E. coli *O15:H7 strain 86-24 [26] (kindly provided by Dr. Rod Moxley from the University of Nebraska, Lincoln-Nebraska) on day 98. Animals were inoculated with one liter of milk replacer containing 10^10 ^CFU *E. coli *O15:H7 strain 86-24 that had been suspended in 50 ml of 5% magnesium trisilicate, 5% sodium bicarbonate, and 5% magnesium carbonate inoculum buffer.

### Collection of fecal samples

Fecal samples from all the calves were collected daily from day 0 to day 42 post-inoculation to determine the level of vaccine strain shedding. After 42 days, fecal samples were collected weekly to determine the presence of *S*. Dublin vaccine strain shedding. Fecal samples were collected daily for 30 days to determine the amount of *E. coli *shedding following oral challenge with *E. coli *O157:H7 strain 86-24 on day 98.

### Collection of peripheral blood for serum IgA measurement and enumeration of intimin-specific IgA-secreting cells

Peripheral blood was collected weekly for serum IgA and ELISpot assays. Blood was collected in serum separator tubes, kept at 37°C for 6 hours, and centrifuged at 2000 rpm for 30 min. Clarified serum was collected and stored at -20°C for serum IgA detection.

### Serum IgA antibody measurement

An Immulon 2-HB flat bottom 96-well microtiter plate (Thermo Labsystems, Franklin, MA) was coated overnight at 4°C with 100 μl/well (5 μg/ml) His-tagged intimin. The plate was washed 3 times with wash buffer (PBS with 0.05% Tween 20), then blocked for 1 h at 37°C with 250 μL/well PBS containing 3% (wt/vol) dried nonfat milk powder (Carnation, Nestle, Glendale, CA). Bovine serum was diluted to 1:1000 with blocking buffer, and 100 μl/well volume of diluted serum was added to the plate and incubated for 3 h at 37°C. Wells were washed 3 times with wash buffer. One-hundred μl rabbit of anti-bovine IgA-horseradish peroxidase conjugate (Bethyl, Montgomery, TX) diluted 1:1000 in wash buffer with 3% (wt/vol) dried nonfat milk powder were added to each well. The plates were then incubated for 1 h at 37°C. Following incubation, the plates were washed 3 times with wash buffer, then incubated with 100 μL/well of a 1:1 mixture of 2,2'-azino-bis(3-ethylbenzthiazoline-6-sulphonic acid) (ABTS) peroxidase substrate and peroxidase solution B (KPL, Gaithersburg, MD) for 1 h at 37°C. After color development (5 min at RT), absorbance was measured at 410 nm on a microplate reader (FLUOstar Optima, BMG Labtechnologies, INC, Durham, NC).

### Mucosal (fecal) IgA antibody measurement

For the detection of mucosal IgA, feces were collected in individual sterile 50 ml centrifuge tubes from all calves. Fecal samples were weighed and liquefied by the addition of ice-cold 0.1 M sodium acetate buffer pH 4.5 (ratio 1:2). This fecal sample-buffer mixture was incubated at 56°C for 30 min. To inactivate proteolytic enzymes, a cocktail of soybean trypsin inhibitor, aprotinin, and phenylmethylsulphonyl chloride (PMSF) was added to this mixture and incubated for 30 min on ice [27]. After incubation, the fecal suspension was centrifuged at 15,000 g at 4°C for 30 min. Clarified supernatant was filtered through a low binding sterile filter (0.45 mM). Titers of intimin-specific IgA in the fecal sample were detected essentially the same way as described above for the intimin-specific IgA in serum, using fecal supernatant instead of serum samples.

### ELISpot assay to detect intimin-specific IgA-secreting cells in peripheral blood

Peripheral blood samples were collected directly into 8 ml BD Vacutainer CPT (Becton Dickinson Vacutainer systems, Franklin lakes, NJ) that contained 1.0 ml of 0.1 M sodium citrate, 1 ml of Ficoll-Hypaque and a gel barrier. Peripheral blood mononuclear cells (PBMC) were isolated as described earlier [28]. Tubes were centrifuged at 3000 RPM for 30 min. The buffy coat containing white blood cells was collected and washed in PBS-citrate. Red blood cells were lysed by incubating the cell suspension in RBC lysis buffer (Analytical Genetic Testing Central, INC. Denver, CO) for 15 min. Cells were washed 2 times with PBS-citrate and finally resuspended in RPMI medium (Gibco BRL, Life Technologies, Inc., Grand Island, NY) supplemented with 15% fetal bovine serum, L-glutamine and sodium pyruvate. Viable cells were counted using trypan blue exclusion dye and a hemacytometer. Cells were kept on ice until they were aliquoted for the ELISpot assay. For the detection of intimin-specfic IgA-secreting cells, individual wells of an ELISpot plate (Millipore Cooperation, 290 Concord Road Billerica, Massachusetts) were incubated at 4°C overnight with 0.5 ug affinity purified bovine IgA diluted in coating buffer (50 mM carbonate buffer, pH 9.6). The coated plate was emptied and rinsed once with supplemented RPMI medium, then blocked with supplemented RPMI media at RT for 3 hrs. For ELISpot assays, 10^5 ^PBMC were plated in duplicate wells. Cells were stimulated with His-tagged intimin (200 ng per well) or PHA (100 ng/well from the Sigma Chemical Company, St. Louis, MO) and incubated for 18 hrs at 37°C in a humidified incubator containing 10% CO_2_. After incubation, cells were rinsed once with distilled water, then washed 3 times with PBS containing 0.05% Tween 20 (PBS-T). Anti-IgA antibodies conjugated to HRPO (100 ul of 1:1000 dilution in PBS-T) were added to each well, and the plate was further incubated for 3 hrs at 37°C. The plate was washed again with PBS-T and spots developed using 100 ul of substrate solution (3-amino-9-ethylcarbazol tablet from Sigma Chemicals, St. Louis, MO, reconstituted as per the manufacturer's recommendation). After development, the plate was emptied and rinsed ten times with distilled water. Antigen stimulated spots were reported by subtracting the number of spots obtained from wells without stimulant from the number of spots obtained in stimulant-added wells.

### Qualitative Salmonella fecal culture

Shedding of *Salmonella *was monitored by collecting daily fecal swabs, followed by enrichment in tetrathionate broth (Beckton Dickinson and Company, Franklin Lakes, NJ), and in Rappaport-Vassiliadis R10 broth (Beckton Dickinson and Company, Franklin Lakes, NJ). Bacteria were enumerated by plating serial dilutions onto XLT-4 plates (Beckton Dickinson and Company, Franklin Lakes, NJ).

### Quantitative *E. coli *O157:H7 fecal culture

Ten g samples of feces were immediately processed in a Stomacher, serially diluted in sterile phosphate-buffered saline, and plated in triplicate onto Sorbitol-MacConkey agar. The sensitivity of the direct plating was 50 CFU/g. A 10 g fecal sample was also added to enrichment broth (Tryptic Soy Broth with 0.15% bile salts), incubated overnight at 37°C, and plated onto selective medium. Colonies isolated on selective medium were confirmed as *E. coli *O157:H7 following the instructions of latex agglutination kit (BD Difco™E. Coli Antisera kit from Becton, Dickinson and Company, Cockeysville, MD).

### Necropsy

Calves were euthanized by captive bolt, and a complete necropsy was performed. At necropsy, tissue samples for bacteriology and histopathology were collected from abomasum, omasum, duodenum, jejunum, ileum, cecum spiral colon, distal colon, rectum and mesenteric lymph node. Homogenates of each tissue were prepared in the Stomacher by mincing two 6 mm biopsy punches of each sample in phosphate-buffered saline. The tissue homogenates were then plated and incubated overnight at 37°C for enumeration of bacteria.

### Statistical analysis

Data were analyzed using SAS version 9.1 (SAS Institute, Cary, NC). Statistical analysis was performed by repeated measures analysis test for between-subject effects (TRT), within-subject effects (Time), and within-subject-by-between-subject interaction effect (TRT*Time). Interaction effects are the joint effects of pairs, triplets, or higher-order combinations of the independent variables, different from what would be predicted from any of the independent variables acting alone. When an interaction is present, the effect of an independent on a dependent varies according to the values of another independent. If the probability of F is less than 0.05 for any such combination, we conclude that the interaction of the combination has an effect on the dependent. Note that the concept of interaction between two independents is not related to the issue of whether the two variables are correlated.

## Results

### Construction of attenuated *Salmonella *strains expressing cloned intimin

**Figure 1:**
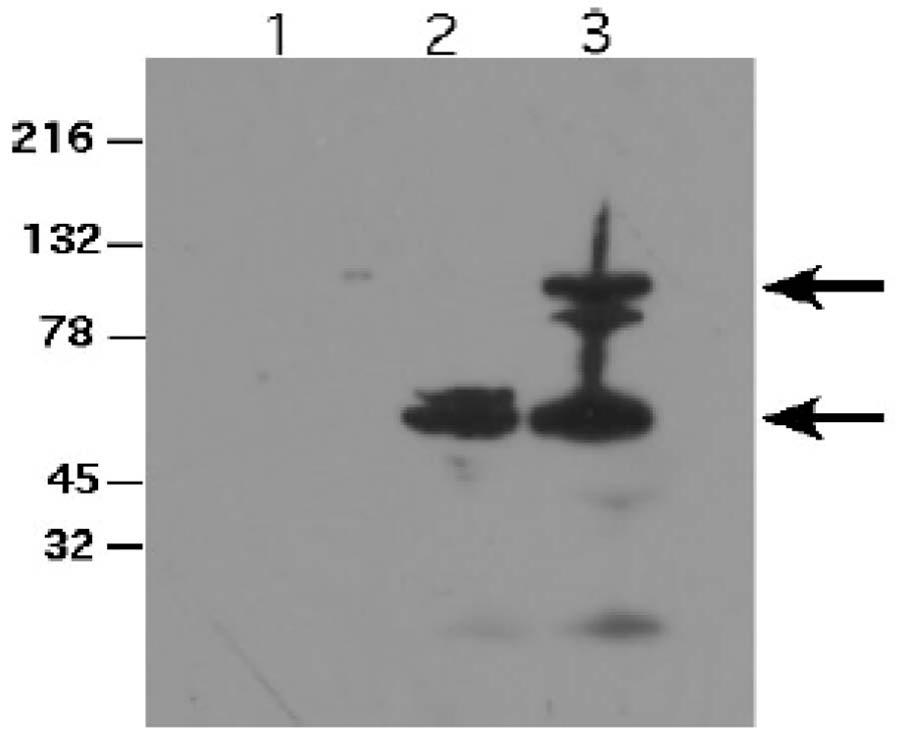
**Expression of Intimin in *aroA *mutant *S*. Dublin**. Protein from overnight cultures of *S*. Dublin *aroA*, *S*. Dublin *aroA *with pRB3::*eaeA*, or *E. coli *K12 with pEB310 were separated on 4-20% SDS-PAGE and expression of intimin determined by western blot. Lane 1, *S*. Dublin *aroA*, lane 2 *S*. Dublin *aroA *with pRB3::*eaeA*, and lane 3, *E. coli *K12 with pEB310. The full-length intimin protein is 96 kD (upper arrow) present in *E. coli *K12 with pEB310 but absent in *S*. Dublin *aroA *with pRB3::*eaeA*. A smaller immunoreactive protein (lower arrow) is present in both *S*. Dublin with pRB3::*eaeA *and *E. coli *K12 with pEB310.

The *eaeA *gene including the upstream promoter region from *E. coli *O157:H7 86-24 was amplified by PCR from pEB310 and cloned into the low copy, RK2-based plasmid pRB3 [29]. This plasmid contains the *par *(partition) locus from RK2 and insures plasmid segregation and stable maintenance even in the absence of selection. This plasmid has been previously used for *in vivo *complementation of *S*. Typhimurium mutations in mice [30,31]. Western blot analysis revealed the production of full length EaeA as well as a smaller ~50-60 kD protein in *E. coli *K12 carrying pEB310. An immunoreactive protein corresponding to the smaller protein was also present in *S*. Dublin *aroA *with pRB3::*eaeA*, but the full length 96 kD EaeA protein was not seen (Figure 1). The presence of the smaller protein in both *E. coli *and *S*. Dublin suggests that EaeA is subject to proteolytic cleavage. A significant quantity of the smaller immunoreactive protein was expressed in *S*. Dublin *aroA *pRB3::*eaeA*, and this strain was used for subsequent vaccine trials.

### Vaccination of calves with *S*. Dublin vaccine strains

**Figure 2:**
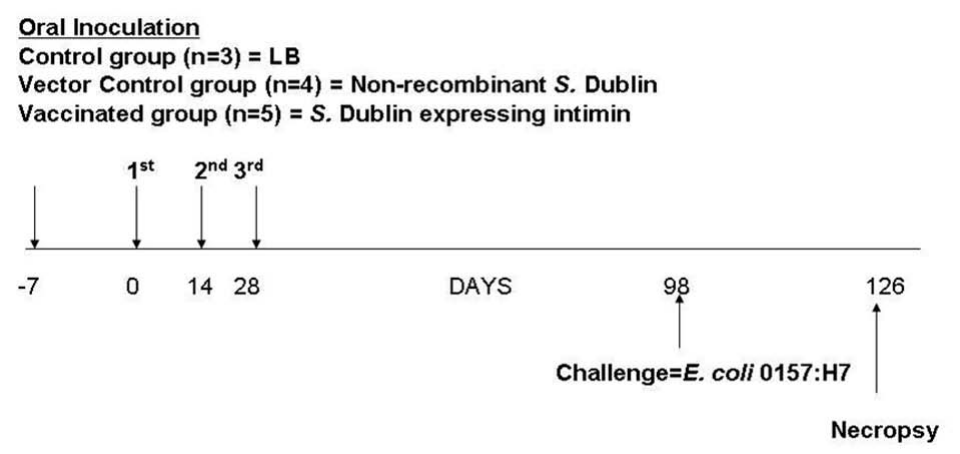
**Time frame of experiment and collection of samples**. Calves were divided into 3 treatment groups. Calves (n = 3) in the control group were fed only milk on days 0, 14 and 28. Calves (n = 4) in the vector group were orally inoculated with the live attenuated non-recombinant *S*. Dublin strain on days 0, 14 and 28. Calves (n = 5) in the vaccinated group were inoculated orally with live attenuated recombinant *S*. Dublin expressing *E. coli *O157:H7 intimin on days 0, 14 and 28. All calves were challenged orally with *E. coli *O157:H7 on day 98 post-vaccination. Fecal samples from calves were collected daily from all calves from day 0 to day 42 post-inoculation for *Salmonella *culture, and fecal samples were collected weekly thereafter. After oral *E. coli *O157:H7 challenge, fecal samples were collected daily to monitor *E. coli *O157:H7 shedding. Peripheral blood samples were collected weekly after inoculation to measure levels of serum IgA and to detect intimin-specific IgA-secreting cells by ELISpot assay.

All calves were orally inoculated with the non-recombinant *S*. Dublin *aroA *vector or with intimin-expressing *S*. Dublin *aroA *on days 0, 14 and 28. A detailed time line for the experiment is provided in Figure 2.

**Figure 3:**
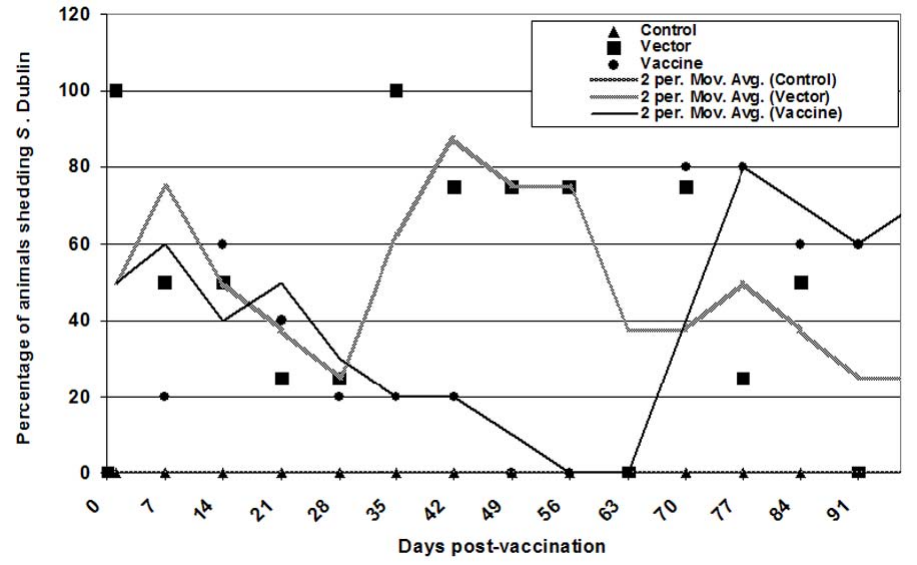
**Duration (x axis) and percentage (y axis) of *S*. Dublin positive calves as measured by qualitative evaluation of fecal shedding**. Calves were inoculated orally as indicated on days 0, 14 and 28. Solid markers (triangle, square or circle) indicate the percentage of calves shedding *S*. Dublin. A moving average trendline was calculated to normalize fluctuations in shedding. The moving average was calculated by setting the period as 2, with the average of the first two data points used as the first point in the moving average trendline. The average of the second and third data points was used as the second point in the trendline, and subsequent trendline points were calculated accordingly.

Calves that received *S*. Dublin strains shed for variable amounts of time following vaccination. Most calves shed the vaccine strains intermittently until the end of the experiment (126 days post-immunization), indicating the establishment of the vector/vaccine strain in the host. Figure 3 depicts fecal shedding of *S*. Dublin strains until 98 days post-immunization. Following the first immunization, both vector and vaccinated groups had similar percentages of calves positive for shedding of *S*. Dublin *aroA *(vector 33%, vaccinated 40%). However, after the third immunization, the vector group contained significantly higher numbers of calves positive for *S*. Dublin shedding as compared to animals immunized with intimin-expressing *S*. Dublin. In order to normalize short-term fluctuations and highlight longer-term trends, we calculated the moving average for the shedding of Salmonella after immunization (Figure 3). By the end of the experiment, the frequency of positive shedders in both the groups was similar (~44%).

### Serum IgA antibody response

**Figure 4:**
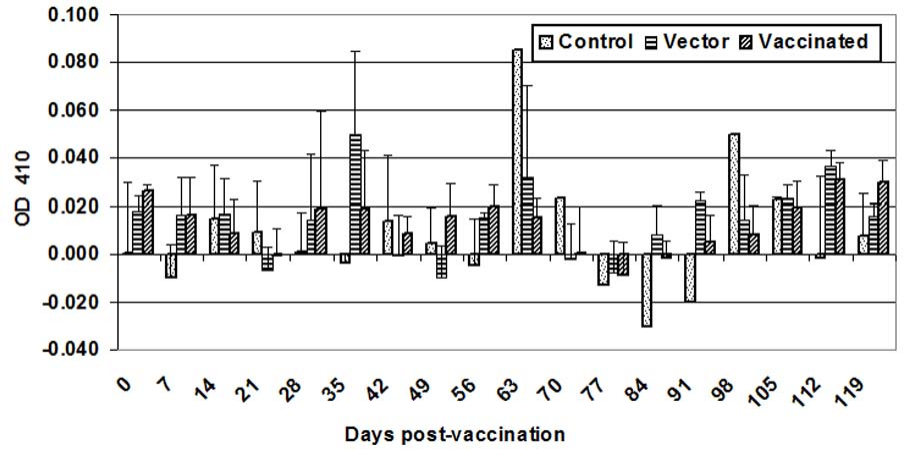
**Serum IgA antibody response**. Peripheral blood was collected weekly from all calves during the length of the vaccination study. Intimin-specific serum IgA antibody concentrations were measured by ELISA. The bar graph depicts the mean (± standard deviation) of animals in each group. Normalization of data was performed by subtracting the OD_410 _of diluent-only wells from experimental values.

**Figure 5:**
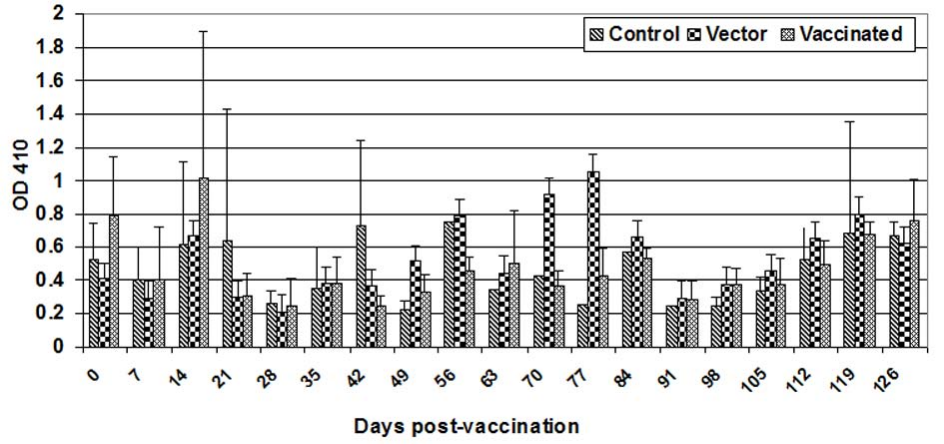
**Mucosal (fecal) IgA antibody response**. Intimin-specific fecal IgA antibody concentrations were measured by ELISA. The bar graph depicts the mean (± standard deviation) of calves in each group. Normalization of data was performed by subtracting the OD_410 _of diluent-only wells from experimental values.

Intimin-specific IgA was measured weekly in the serum of all calves throughout the experiment (Figure 4). We observed an increase in intimin-specific IgA in calves receiving either the vector or the intimin-expressing strain. Statistical analyses revealed a significant time effect (P = 0.036), but no significant treatment effect (P = 0.87) or treatment × time interaction (P = 0.4). Also, prior to the *E. coli *O157:H7 challenge, there was a significant time effect (P = 0.0009), but no significant treatment effect (P = 0.25) or treatment × time interaction (P = 0.26). A sporadic increase in intimin-specific IgA levels was observed in the control group. However, we did not notice any symptoms of clinical infection in these animals. Mucosal (fecal) IgA responses with intimin-specific IgA antibody were observed in all animals (Figure 5). There was a significant time effect (P < 0.001), but no significant treatment effect (P = 0.2) or treatment × time effect (P = 0.5). Fecal anti-intimin IgA levels prior to the *E. coli *O157:H7 challenge showed a significant time effect (P < 0.001) and treatment × time interaction (P = 0.0009), but no significant treatment effect (P = 0.2).

### Intimin-specific IgA secreting cells in peripheral blood mononuclear cells

**Figure 6:**
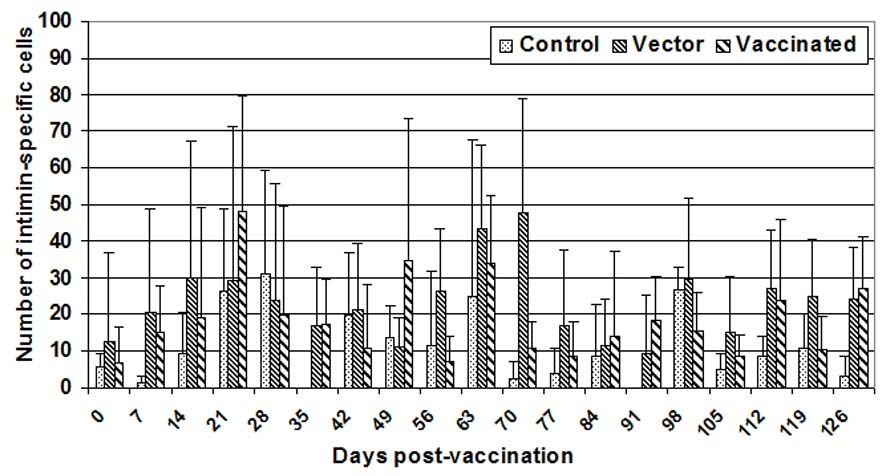
**Intimin specific IgA secreting cells in peripheral blood mononuclear cells**. 10^5 ^PBMC were plated on bovine IgA-coated wells. Cells were stimulated with intimin (200 ng per well) and incubated for 18 hrs at 37°C. After incubation, cells were incubated with anti-IgA antibody conjugated to HRPO, and spots were developed using substrate solution. Intimin-specific IgA-secreting cells were determined by subtracting the number of spots obtained in wells without any stimulant from the number of spots obtained in wells to which intimin was added. The bar graph depicts the mean (± standard deviation) of animals in each group.

An increase in intimin-specific IgA secreting cells was measured by the ELISpot assay (Figure 6). There was a significant treatment effect (P = 0.04) and time effect (P = 0.001), but no significant treatment × time interaction (P = 0.19). Using multiple comparisons, there was a significant difference between control and vaccinated animals, and between control and vector animals. However, before the *E. coli *O157:H7 challenge, there was a significant time effect (P < 0.03) and treatment × time interaction (P = 0.03), but no significant treatment effect (P = 0.6).

### Shedding of *E. coli *O157:H7

**Figure 7:**
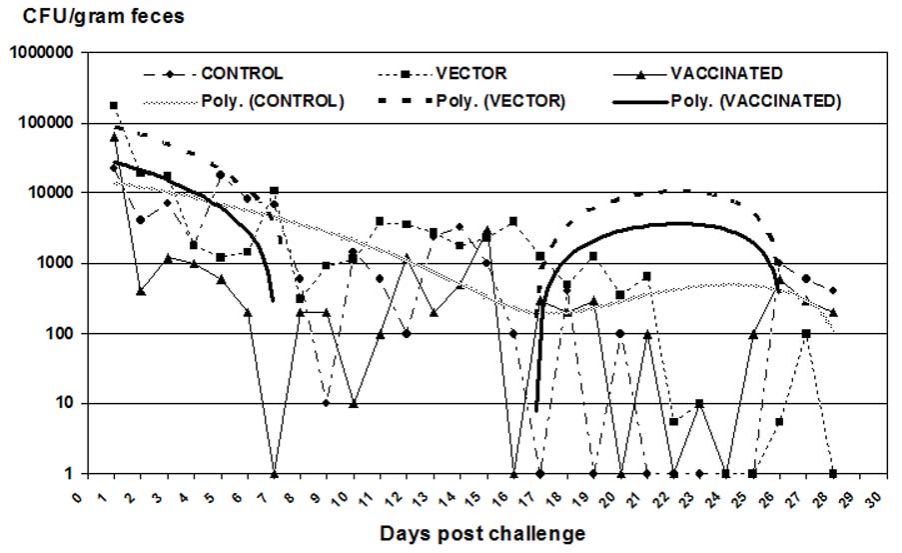
**Magnitude and duration of fecal shedding of E. coli O157:H7**. Magnitude (median of each group, CFU/gram of feces) and duration of fecal shedding post-challenge with *E. coli *O157:H7 were calculated quantitatively by direct plating as well as by enrichment culture following oral challenge with *E. coli *O157:H7 during the 30 days post challenge. Specimens containing less than the detection limit (*E. coli *O157:H7 found only by enrichment) were assigned a value of 10. Negative specimens were assigned a value of 1. Samples too numerous to count (TNTC) were considered to contain the maximum number counted.

All calves were challenged orally with *E. coli *O157:H7 on day 98 post-vaccination. *E. coli *O157:H7 shedding was measured after the challenge (Figure 7). There was a significant time effect (P < 0.001) and treatment × time interaction (P = < 0.001), but no significant treatment effect (P = 0.27). However, when the trend of shedding *E. coli *O157:H7 was calculated as a polynomial trendline, the trend revealed that the vaccinated calves descended into the "valley" of the trendline earlier than those who received the vector, whereas the control group never reached the valley of the trendline during the entire study period. Moreover, levels of shedding were lower in animals receiving the intimin-expressing vaccine strain. This indicates an early, albeit transient, clearance of the challenge strain in vaccinated calves.

### Bacteriology and Histopathology

None of the examined tissue was positive for the colonization of bacteria. No notable differences were detected in the histopathology among the treatment groups.

## Discussion

Enterohemorrhagic *Escherichia coli *(EHEC) such as strain O157:H7 is an etiologic agent of acute enteric diseases in both humans and neonatal calves [32]; however, mature cattle are not affected. *E. coli *O157:H7 can enter the human food supply from cattle via fecal contamination of beef carcasses at slaughter [33]. Intimin is an outer membrane protein expressed by several human and animal enteric pathogens, including enteropathogenic *E. coli *and EHEC [34-38]. Antibodies to intimin may prevent the initial steps of EHEC colonization in the gastrointestinal tract [39-41]. Anti-intimin immune responses can modulate the outcome of experimental infection with the EPEC-like bacterium *Citrobacter rodentium *in rabbits and supports the inclusion of intimin as a component of an EPEC or EHEC vaccine [42]. Vaccination of cattle has significant potential as a pre-harvest intervention strategy to reduce *E. coli *O157:H7 shedding. However, the ability of intimin to elicit protective immune responses in the bovine intestinal tract has not previously been demonstrated. We hypothesized that the mucosal immune response elicited by live attenuated *Salmonella enterica *serovar Dublin expressing the intimin protein of *E. coli *O157:H7 would reduce the magnitude and duration of *E. coli *O157:H7 colonization and fecal shedding.

In the present study, orally-administered *S*. Dublin vector or *S*. Dublin expressing *E. coli *O157:H7 intimin were recovered for up to 98 days post-inoculation in the feces of calves, confirmed the establishment of intestinal carriage. The frequency of positive *S*. Dublin shedders in both vaccinated groups at the end of experiment were similar as observed in other studies in which vaccine strains administered to cattle were shed for a considerable period of time [31]. From 35-70 days post-inoculation, the *S*. Dublin vector control strain was detectable in a significantly higher proportion of calves than the intimin-expressing *S*. Dublin vaccine strain. Levels of intimin-specific IgA in serum and feces were not significantly higher in calves receiving intimin-expressing *S*. Dublin. However, cattle immunized with the intimin-expressing strain group exhibited a reduced magnitude and duration of *E. coli *O157:H7 shedding following oral challenge.

Earlier studies reported that infection of seropositive adult cattle with *E. coli *O157:H7 increases serum antibody titers to intimin and to the translocated intimin receptor (Tir) [23]. Intimin interacts not only with Tir, but also with host cell intimin receptor(s) on the luminal surface of intestinal epithelia, including integrin and nucleolin [43,44]. These receptors are potentially accessible as binding sites for intimin during vaccination with recombinant *S*. Dublin. While antibodies directed against either Tir or intimin might impede intimin-Tir interactions, antibodies to intimin might be anticipated to inhibit EHEC binding to alternative host receptors as well. Recently, it has been shown that vaccination with a combination of antigens associated with type III secretion system-mediated adherence; the translocon filament protein, EspA, the extracellular region of the outer membrane adhesin, intimin, and Tir significantly reduced shedding of EHEC O157 from experimentally infected animals [45].

In our study, initial shedding of *E. coli *O157:H7 after challenge was comparable in the groups receiving either vector or intimin-expressing *S*. Dublin, and significantly lower than in the control group. Earlier studies have indicated that *E. coli *persists (a challenge dose of 10^9^) for days to weeks in the bovine intestinal tract before being cleared [46]. Similar results were observed in this study; however, it is important to note that the challenge dose in the present study was higher (10^10^). The polynomial trendline revealed that *E. coli *O157:H7 was cleared more rapidly from vaccinated calves than from control or vector-vaccinated animals. This provides evidence in support of the principle that potentiation of immune responses to intimin at the mucosal surface can reduce shedding of the pathogenic *E. coli *O157:H7 strain. Also, the possibility of interaction of various components of adaptive immunity due to initial Salmonella (vector) infection could not be ruled out [47]. We originally hypothesized that the protective responses would be related to fecal concentrations of intimin-specific IgA. However, the mechanism of vaccine protection is clearly more complex, as anti-intimin fecal IgA levels did not correlate with fecal shedding. Enteric mucosal IgA responses against intimin and type III secreted proteins were identified in rectal mucus and in the rectal tissue respectively [48,49]. These studies definitely indicate the importance of other clinical samples (tissue and rectal mucus) for studying the mucosal immune response. Moreover, protection against enteric pathogens by immunization does not essentially require secretory IgA [45], and intestinal clearance of intimin-expressing *Citrobacter rodentium *has been shown to require B cells and IgG antibodies, but not secretory IgA [45]. Importantly, intimin-specific antibody titers in colostrum and serum of dams were found to be increased after parenteral vaccination with intimin [50]. In another study, immunization of calves with the cell-binding domain of intimin subtypes beta or gamma via the intramuscular route induced antigen-specific serum IgG1 and, in some cases salivary IgA responses, but did not reduce the magnitude or duration of faecal excretion of EHEC upon subsequent experimental challenge [51]. The role of IgG in intimin-expressing vaccine induced protection of calves is worthy of further investigation.

Of note, reduction in colonization and shedding was obtained in this study by oral vaccination without a preceding parenteral inoculation. However, oral vaccines can significantly boost mucosal immune responses when primed by parenteral vaccine administration [52,53]. Parenteral priming of the immune system facilitates the gut associated lymphoid tissue to react more rapidly to antigens delivered by oral immunization, and may decrease the likelihood of inducing oral immune tolerance [24]. Another possible explanation for the modest degree of reduction in colonization and shedding of *E. coli *O157:H7 may be that intimin-specific mucosal IgA was already present prior to immunization. Thus, this antibody may have interfered with ability of the intimin-expressing *S*. Dublin vaccine strain to effectively reach gut-associated lymphoid tissue and augment local immune responses. One limitation to the present study is the need for replication in outbred populations of cattle having a more defined immune status.

## Conclusions

In summary, a live *S*. Dublin vaccine strain expressing the *E. coli *O157:H7 intimin protein effectively colonized the intestines of calves after vaccination. Immunization resulted in a transient clearance and subsequently reduced colonization and shedding of *E. coli *O157:H7 following challenge.

## Authors' contributions

SK coordinated sampling of animals, completed immunological assays, analyzed data and drafted the manuscript. SZ cultured *S*. Dublin. FCF and SJL designed and constructed the *S*. Dublin vaccine strain. DH and RP isolated PBMC from blood and evaluated shedding of bacteria. WA performed statistical analyses. LGA, FCF, and SJL are funded co-applicants who designed the study and edited the manuscript. All authors read and approved the final manuscript.
